# Cancer Mechanisms and Emerging Therapies

**DOI:** 10.3390/pharmaceutics13071045

**Published:** 2021-07-09

**Authors:** Diana Gulei, Alice Indini, Carmen Jerónimo, Cristina-Adela Iuga, Francesco Grossi

**Affiliations:** 1 MEDFUTURE—Research Center for Advanced Medicine, “Iuliu-Hatieganu” University of Medicine and Pharmacy, Marinescu 23 Street/Louis Pasteur 4–6 Street, 400337 Cluj-Napoca, Romania; diana.c.gulei@gmail.com; 2 Medical Oncology Unit, Fondazione IRCCS Ca’ Granda Ospedale Maggiore Policlinico, 20122 Milan, Italy; alice.indini@gmail.com; 3 Cancer Biology and Epigenetics Group, Research Center of IPO Porto (CI-IPOP)/RISE@CI-IPOP (Health Research Network), Portuguese Oncology Institute of Porto (IPO Porto)/Porto Comprehensive Cancer Center (Porto.CCC), R. Dr. António Bernardino de Almeida, 4200-072 Porto, Portugal; carmenjeronimo@ipoporto.min-saude.pt; 4 Department of Pathology and Molecular Immunology, Institute of Biomedical Sciences Abel Salazar, University of Porto (ICBAS-UP), Rua Jorge Viterbo Ferreira 228, 4050-513 Porto, Portugal; 5 Department of Pharmaceutical Analysis, Faculty of Pharmacy, “Iuliu Hațieganu” University of Medicine and Pharmacy, Louis Pasteur Street 6, 400349 Cluj-Napoca, Romania; 6 Medical Oncology Unit, Department of Medicine and Surgery, University of Insubria, ASST dei Sette Laghi, 21100 Varese, Italy; francesco.grossi@asst-settelaghi.it

Over the last decades, cancer has become one of the most relevant health issues at a worldwide level. Its constantly rising incidence is estimated to exceed the current rates by 47% until 2040, according to Global Cancer Statistics 2020, thus representing a considerable burden at both the economic and social level in many countries. The decisive factor in terms of cancer clinical management consists of the mortality rates that reflect the current translational research capacity and availability of new and efficient therapeutics that can surpass the heterogenous, adaptable and dynamic profile of malignant pathologies. Indeed, cancer is slowly surpassing heart diseases in terms of leading causes of death, being now the first in line for premature mortality in 57 countries, second in 55 countries and third–forth in 23 countries (World Health Organization 2020).

In terms of translational oncology research, one of the main issues in treating cancer is heterogeneity, which makes the idea of one size fits all limited and unable to provide successful therapeutic results. With growing knowledge on the mechanisms of cancer development, anti-cancer therapies have evolved greatly to include drugs aimed toward the immune system, targeted therapy, gene editing, epigenetic agents, and many others. At the base of these therapeutic achievements are the biochemical processes and signaling pathways that are aberrantly expressed in cancer cells as well as in the tumor microenvironment.

This Special Issue brings together a series of articles highlighting the current challenges and complementary therapeutic opportunities for the treatment of cancer and also provides an overview of alternative medicine and naturally-derived therapeutic compounds, specific targeting of oncogenic proteins and microRNA modulation for the restoration of homeostatic expression. 

Lobo et al. [1] present the effects of a synthetic flavonoid, MLo1302, in the context of a testicular germ cell tumor. This compound is designed to target the DNA methyltransferase enzyme responsible for the abnormal methylome in malignant pathologies including testicular germ cell cancer and the subsequent repression of the gene expression. In vitro testing showed that MLo1302 inhibits the tumor cell viability, stimulates apoptosis and cytotoxicity and concomitantly reduces the cell proliferation. The functional effects of MLo1302 were validated also at the molecular level by the identification of the deregulated expression of the genes responsible for apoptosis induction. In light of these results, the authors propose novel treatment options for testicular germ cell tumors that are associated with less toxic side effects compared with the current standard of care (i.e., platinum-based chemotherapy).

Ding et al. [2] approach the idea of affibody molecules conjugated with drugs targeting the human epidermal growth factor receptor 2 (HER2), designed to enhance drug delivery to tumor cells and reduce systemic toxicity. In vitro testing showed an increased toxicity of affibody carriers conjugated with cytotoxic mcDM1 in cancer cell lines with a high expression of HER2 while minimal toxicity was observed in cell lines with a medium or low expression of HER2. Further studies on animal models bearing ovarian adenocarcinoma xenografts (SKOV3 cells) showed an increased drug concentration at the tumor by 1.45-fold; however, this advantage was partially compromised by a higher non-specific drug accumulation in the liver, spleen and bone sites. The authors highlight the importance of further investigation on the design of the therapeutic complex to preserve the increased drug accumulation at the tumor but protect at the same time the normal tissue from excessive drug uptake. 

Chizenga et al. [3] present the complex blueprint of cancer and the beneficial role of complementary and alternative medicine (CAM) therapies in the context of cervical cancer. The authors highlight the spectrum of adverse events during conventional oncological therapies (i.e., chemotherapy and radiation therapy) where complete disease remission is often associated with a physiological imbalance due to the toxicity of the cancer treatments. Therefore, the authors’ proposal is to integrate CAM therapies with allopathic medicine to enhance the anti-cancer properties of both strategies and also obtain a physiological normalcy post-therapy. The article also comprehensively presents the standard treatment and novel therapeutic strategies currently in use for cervical cancer as well as peculiar features of cervical cancer such as genomic instability, intracellular signaling, the mechanisms of immune evasion, angiogenesis and the interplay with the tumor microenvironment, which can be hijacked through integrative medicine for the benefit of the overall health of patients.

Indini et al. [4] present the current therapeutic options to target the RAS family proteins, which present a high mutational frequency in all human cancers and are associated with driver roles in tumorigenesis. The authors comprehensively assess the current status of drugs targeting the Kirsten RAS (KRAS) protein. Despite being historically known as “undruggable” due to specific conformations not allowing the interaction with small inhibitory molecules, advances in oncological research have led to the development of KRAS inhibitors, namely, drugs targeting the KRAS G12C isoform in non-small cell lung cancers. The article also presents data of KRAS inhibitors for the treatment of other KRAS-mutated solid tumors, specifically colorectal cancer, low-grade serous ovarian carcinomas, pancreatic cancer and endometrial cancer and provides an updated overview of clinical trials with KRAS inhibitors currently ongoing. 

Cenariu et al. [5] attempt to decipher the therapeutic role of micro-ARN-125b in colon cancer, a role that has been presented as controversial in the literature, with both evidence of tumor suppressor activity and reports of a possible oncogenic role. Through in vitro and molecular assays, the authors demonstrate that micro-ARN-125b preferentially functions as a therapeutic molecule (through exogenous upregulation) only in TP53-mutant colon cancer cells through the inhibition of an oncogenic TP53 mRNA. On the contrary, miRNA replacement is not associated with anti-cancer benefits in TP53 wild-type cells in which this protein plays inhibitory roles on the tumorigenesis. 

We hope that this Special Issue will help readers in their research work providing insights into cancer mechanisms and current evolving therapies. We want to express our appreciation for the authors of the Special Issue that contributed with quality manuscripts and toward the reviewers and editorial office that critically revised the work of the researchers. Last but not least, we recognize the contribution of Managing Editor Sandy Cheng who was significantly involved in all steps of the preparation of the current Special Issue. 

## References

[1] Lobo J., Cardoso A.R., Miranda-Gonçalves V., Looijenga L.H.J., Lopez M., Arimondo P.B., Henrique R., Jerónimo C. (2021). Targeting Germ Cell Tumors with the Newly Synthesized Flavanone-Derived Compound MLo1302 Efficiently Reduces Tumor Cell Viability and Induces Apoptosis and Cell Cycle Arrest. Pharmaceutics.

[2] Ding H., Xu T., Zhang J., Tolmachev V., Oroujeni M., Orlova A., Gräslund T., Vorobyeva A. (2021). Affibody-Derived Drug Conjugates Targeting HER2: Effect of Drug Load on Cytotoxicity and Biodistribution. Pharmaceutics.

[3] Chizenga E.P., Abrahamse H. (2021). Biological Therapy with Complementary and Alternative Medicine in Innocuous Integrative Oncology: A Case of Cervical Cancer. Pharmaceutics.

[4] Indini A., Rijavec E., Ghidini M., Cortellini A., Grossi F. (2021). Targeting KRAS in Solid Tumors: Current Challenges and Future Opportunities of Novel KRAS Inhibitors. Pharmaceutics.

[5] Cenariu D., Zimta A.A., Munteanu R., Onaciu A., Moldovan C.S., Jurj A., Raduly L., Moldovan A., Florea A., Budisan L. (2021). Hsa-miR-125b Therapeutic Role in Colon Cancer Is Dependent on the Mutation Status of the TP53 Gene. Pharmaceutics.

